# An advanced PdNPs@MoS_2_ nanocomposite for efficient oxygen evolution reaction in alkaline media†

**DOI:** 10.1039/d3ra04738e

**Published:** 2023-11-03

**Authors:** Umair Aftab, Muhammad Yameen Solangi, Aneela Tahira, Abdul Hanan, Muhammad Ishaq Abro, Amal Karsy, Elmuez Dawi, Muhammad Ali Bhatti, Riyadh H. Alshammari, Ayman Nafady, Alessandro Gradone, Raffaello Mazzaro, Vittorio Morandi, Antonia Infantes-Molina, Zafar Hussain Ibupoto

**Affiliations:** a Department of Metallurgy and Materials Engineering, Mehran University of Engineering and Technology 76080 Jamshoro Pakistan; b Institute of Chemistry, Shah Abdul Latif University Khairpur Mirs Sindh Pakistan; c Dr. M. A. Kazi Institute of Chemistry University of Sindh Jamshoro 76080 Sindh Pakistan zaffar.ibhupoto@usindh.edu.pk; d Key Laboratory of Superlight Material and Surface Technology, Ministry of Education, College of Materials Science and Chemical Engineering, Harbin Engineering University 150001 Harbin PR China; e Department of Inorganic Chemistry, Crystallography and Mineralogy, (Unidad Asociada al ICP-CSIC), Faculty of Sciences, University of Malaga Campus de Teatinos 29071 Malaga Spain; f CNR IMM Via Piero Gobetti 101 40129 Bologna Italy; g Department of Physics and Astronomy, University of Bologna Via Berti Pichat 6/2 40127 Bologna Italy; h Department of Chemistry, College of Science, King Saud University Riyadh 11451 Saudi Arabia; i Nanotechnology Research Centre (NTRC), The British University in Egypt (BUE) Cairo Egypt; j Nonlinear Dynamics Research Center (NDRC), Ajman University Ajman P.O. Box 346 United Arab Emirates; k Institute of Environmental Sciences, University of Sindh Jamshoro Jamshoro 76080 Sindh Pakistan

## Abstract

In response to the increasing availability of hydrogen energy and renewable energy sources, molybdenum disulfide (MoS_2_)-based electrocatalysts are becoming increasingly important for efficient electrochemical water splitting. This study involves the incorporation of palladium nanoparticles (PdNPs) into hydrothermally grown MoS_2_*via* a UV light assisted process to afford PdNPs@MoS_2_ as an alternative electrocatalyst for efficient energy storage and conversion. Various analytical techniques, including scanning electron microscopy (SEM), transmission electron microscopy (TEM), X-ray diffraction (XRD), X-ray photoelectron spectroscopy (XPS), and energy dispersive spectroscopy (EDS), were used to investigate the morphology, crystal quality, and chemical composition of the samples. Although PdNPs did not alter the MoS_2_ morphology, oxygen evolution reaction (OER) activity was driven at considerable overpotential. When electrochemical water splitting was performed in 1.0 M KOH aqueous solution with PdNPs@MoS_2_ (sample-2), an overpotential of 253 mV was observed. Furthermore, OER performance was highly favorable through rapid reaction kinetics and a low Tafel slope of 59 mV dec^−1^, as well as high durability and stability. In accordance with the electrochemical results, sample-2 showed also a lower charge transfer resistance, which again provided evidence of OER activity. The enhanced OER activity was attributed to a number of factors, including structural, surface chemical compositions, and synergistic effects between MoS_2_ and PdNPs.

## Abstract 2

A palladium nanoparticle-decorated MoS_2_ layered structure for the efficient oxygen evolution reaction.

## Graphical abstract

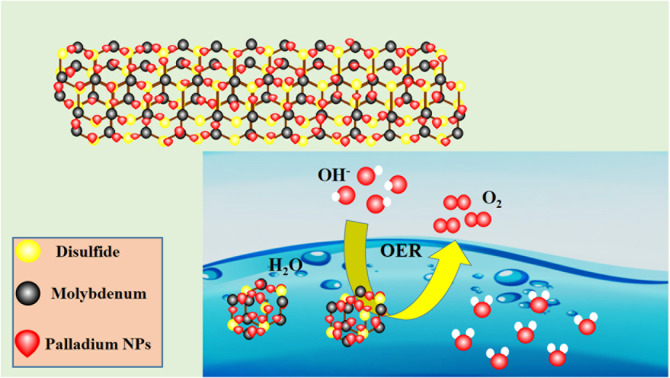

## 1. Introduction

Technological advances, as well as the impact of the industrial revolution on humans and the planet, create new challenges for our well-being. During the past few decades, rapid industrial development has resulted in the greenhouse gas effect being one of the major concerns. Greenhouse gases are primarily generated by the purification and use of fossil fuels, including coal, petrochemical compounds, and natural gas. This raises significant concerns about the feasibility of initiating extensive research on renewable energy reservoir development and related technologies.^1–3^ Hydrogen has high energy density and zero carbon emissions into the atmosphere, so it has shown high potential and is capable of meeting the challenges of the energy sector. Natural gas reservoirs have traditionally been converted to hydrogen *via* steam-reforming. Electrochemical water splitting is a green and cost-effective technology for producing hydrogen to meet energy needs for sustainable and environmentally friendly futures.^4–6^ The electrochemical water splitting process requires electric power, which is generated *via* solar panels or wind generators, which are considered sustainable technologies. Water splitting involves two half-cell reactions, one of which is the hydrogen evolution reaction (HER) and the other is the oxygen evolution reaction (OER). In either case, water splitting is a non-spontaneous reaction and it is accompanied by the use of external energy. However, by using an electrocatalyst as either a cathode or anode, this energy barrier can be overcome.^7^ It has a high energy barrier, making OER half-cell reactions kinetically sluggish compared to HER, therefore, it is not possible to exploit the maximum hydrogen generation from water splitting due to the lack of efficient OER reactions. In order to increase the efficiency of the OER half-cell reaction kinetics, electrocatalysts would be highly effective in reducing the overpotential needed for water splitting, and therefore the activation energy could be lowered.^8–10^ Electrocatalysts based on precious metals, such as iridium (IrO_2_) and ruthenium (RuO_2_), offer efficient OER activity, but their scarcity and cost limit their use on a large scale. The development of low cost, simple, and high-stability electrocatalysts would allow the water splitting process to be adapted to scale up applications. The immediate focus is therefore on nonprecious electrocatalysts, resulting in vigorous research in the last 20 years for more efficient electrocatalysts that possess a minimum amount of noble metals in their compositions.^3,11^ Several materials have been studied for various electrochemical applications, including conductive polymers, carbon derivatives, metal oxides, and metal sulfides. Although transition metal oxides, sulphides, and conductive polymers exhibit redox properties, their industrial applications are restricted by their limited capacitance, low specific surface area, and poor electrical conductivity.^5,12^ The development of energy storage and conversion systems has recently been influenced by the unique characteristics of metal sulfides, including their abundance, low cost, significant electrical conductivity, high theoretical capacitance, ease of preparation, and environmental friendliness.^13^ There has been tremendous interest in two-dimensional (2D) layered dichalcogenides due to their unique characteristics, such as enriched active sites, large surface area, and high ionic conductivity.^14^ Among them, molybdenum disulfide (MoS_2_) is highly investigated because of its high capacitance, catalytic sites, earth abundance, cost effectiveness, and high charge carrying capability.^15^ As with MoS_2_, Mo atoms are located between two layers of S atoms in a sandwich-like structure. Moreover, MoS_2_ possesses three different crystal phases, namely trigonal (1T), hexagonal (2H) and rhombohedral (3R). Compared to two other phases of MoS_2_, the 2H phase is highly stable. In MoS_2_, the 2H and 3R phases are semiconducting materials, whereas the 1T phase is metallic in nature. A heat treatment can change a 3R phase into a 2H phase.^16^ The presence of many metallic oxidation states in MoS_2_ makes it a redox material and an electrocatalyst.^17^ There has been evidence showing that MoS_2_ HER performance is poor due to a lack of unsaturated edges as active sites and also poor electrical conductivity.^18–20^ MoS_2_ has been etched by H_2_O treatment,^21^ treated with NaClO,^22^ treated with oxygen plasma,^23^ and annealed with H_2_ (ref. 24) to accelerate its HER activity. By doping metal or nonmetal into MoS_2_, the basal plane is activated, which enhances the HER performance^25–27^ due to local electron density variation around Mo and S atoms. Through the modulation of electron density of MoS_2_, 3d transition metals (Co, Ni) of the doping agents have proven effective for enhancing HER and OER activities.^26,28,29^ MoS_2_ has been found to have metal sites at its basal panes that are inert towards hydrogen/oxygen reactive species, resulting in poor catalytic performance.^30,31^ It is therefore highly desirable to redesign and reconstruct MoS_2_ basal plane in order to demonstrate efficient OER kinetics through efficient adsorption/desorption.^32–34^ In light of these challenges regarding the development of an efficient MoS_2_ electrocatalyst, we propose the use of palladium (Pd) nanoparticles incorporation into MoS_2_ during the UV light environment for the first time in the literature. The UV light based for the decoration of MoS_2_ nanostructures with Pd is simple, ecofriendly and scalable, hence such fabrication strategies for the design of high performance electrocatalysts are highly desirable. Pd is a noble metal with a high catalytic activity for OER. Noble metals are known for their high conductivity and chemical stability, which makes them ideal for electrocatalytic applications. Pd is particularly active for OER, due to its ability to easily undergo oxidation and reduction reactions. Furthermore, Pd can be easily dispersed on the surface of MoS_2_. MoS_2_ is a layered material with a large surface area, which makes it a good support material for Pd nanoparticles. Pd doping is a promising strategy for improving the performance of MoS_2_ for OER.^35,36^ It can help to increase the activity, stability, and conductivity of the catalyst, which makes it a more attractive option for commercial applications. As a result of enriched unsaturated edge sites, multivalent Mo atoms and synergetic effect, Pd based MoS_2_ composite outperformed OER.

As described here, MoS_2_ nanosheets were synthesized by hydrothermal process followed by Pd combination by a UV light assisted method. A variety of Pd contents were applied to MoS_2_ and their effects on structural changes, chemical composition, and catalytic performance were studied.

## 2. Experimental section

### 2.1. Hydrothermal synthesis of MoS_2_ nanostructures followed by UV light assisted deposition of palladium nanoparticles

**Scheme 1:**
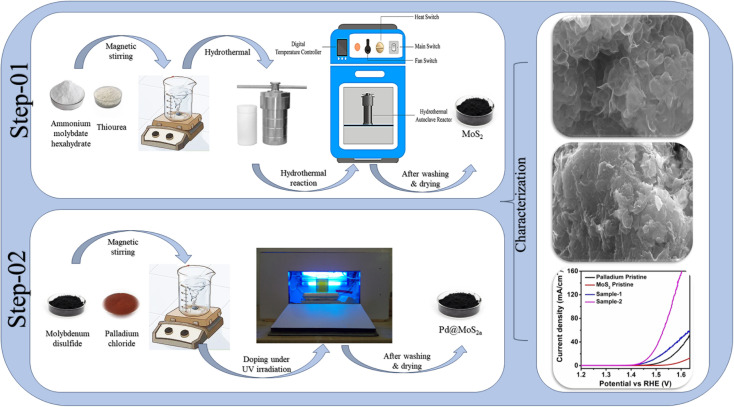
Schematic view of palladium nanoparticles deposited on MoS_2_ nanostructures.

Thiourea (CH_4_N_2_S), ammonium molybdate hexahydrate (H_20_MoN_2_O_10_), palladium chloride (PdCl_2_), hydrochloric acid (HCl), and sulfuric acid (H_2_SO_4_) were purchased from Sigma Aldrich, Karachi, Pakistan. Two sessions were required to synthesize Pd based MoS_2_ nanocomposite. As a first step, MoS_2_ nanostructures were synthesized using a hydrothermal process. Herein, ammonium molybdate hexahydrate of 245 mg and thiourea of 225 mg were dissolved in 70 mL of deionized water. In the next step, the solution was transferred to a Teflon-lined stainless-steel autoclave with a capacity of 100 mL and kept in an electric oven at 210 °C for 20 hours. The autoclave was cooled at room temperature (RT) after the reaction was completed. The nanostructured material was collected from the autoclave. A filter paper was used to remove impurities after several washes with deionized water. Afterwards, the nanomaterial was dried at 100 °C for 3 hours. Second, Pd based MoS_2_ nanocomposites were developed. MoS_2_ nanomaterial was added to three separate beakers of 100 mL deionized water with PdCl_2_ solution (20 mg PdCl_2_ and 0.02 M HCl in 20 mL DI water) of 6 mL, 12 mL and 18 mL. The samples were labelled as sample-1, sample-2 and sample-3. Following sonication for 30 minutes, the solution-containing beaker was placed in an ultraviolet irradiation box for 2 hours under continuous stirring. An irradiation mechanism was used here to convert Pd ions into Pd nanoparticles. The reduction of Pd^2+^ as followed when MoS_2_ was the substrate. Once MoS_2_ was exposed to radiation, electrons would dissociate from holes in the valence band and be released to the conduction band because of the semiconductor nature of MoS_2_ nanostructure. These free electrons were taken up by the PdCl_2_^−^ ions when they approached the MoS_2_ surface. As a consequence, precursors of Pd nanoparticles were created by reducing Pd^2+^ ions. Due to their confined surface plasmon resonance, these precursors would facilitate the absorption of visible light. Furthermore, the presence of Pd nanoparticles on the MoS_2_ nanostructure may stop from recombining of electron–hole pairs on MoS_2_. As a result, additional photogenerated electrons were generated and more Pd^2+^ ions were converted into Pd^0^, growing on the Pd precursors to form giant Pd nanoparticles.^37–40^ Hence, MoS_2_ nanostructures were combined with Pd nanoparticles. Following the combination process, samples were removed from UV irradiation boxes and repeatedly washed with DI water. We collected samples with filter paper and dried them at 100 °C for two hours. As a result, a nanocomposite material was achieved (Scheme 1).

### 2.2. Physical characterizations

A powder X-ray diffractometer (Philips PANanalytical) operated at 45 kV and 45 mA with CuKα radiations of 1.5418 Å was used to examine crystal orientation and phase purity of synthesized samples. A quantitative analysis of phase was carried out with the help of High Score Plus software. XPS analysis of prepared samples was performed on Scienta ESCA 200 Spectrometer at low operating pressure (10^−10^ mbar) and under ultrahigh vacuum using monochromatic X-ray source Al (k-alpha) of photons (1486.6 eV). As a result of the XPS measurement, a 0.65 eV Auf7/2 line of full width at half maximum was observed. Nanostructures were analyzed *via* scanning electron microscopy using a ZEISS Gemini SEM 500 model. High-magnification images of nanostructures were captured with a high-resolution transmission electron microscope (FEI Tecnai F20) equipped with a Schottky emitter at 120 kV. Further, an angle annular dark field detector equipped with scanning transmission electron microscopy (STEM-HAADF) was used to discriminate between samples with varying atomic weights.

### 2.3. Electrochemical measurements for OER activity

VERSASTAT 4-500 Potentiostat was used to measure the electrochemical activity of OER using three electrode cells, namely reference electrode (Ag/AgCl), counter electrode (Pt wire), and working electrode (glassy carbon electrode). First, the catalyst solutions from different samples were prepared by mixing 5 mg of sample with 1 mL of deionized water in a 5 mL bottle. Afterward, 30 microliters of Nafion 5 wt% was added to the solution and homogenized with a sonicator. Alumina slurry was used to clean the working electrode (GCE) *via* polishing-on-polishing cloth. Drop casting the prepared catalyst ink onto the GCE after it was cleaned and dried was then performed. Oxygen evolution reactions were measured in 1.0 M KOH electrolyte solution using linear sweep voltammetry at 5 mV s^−1^ scan rate. To calculate electrochemical active surface area at different scan rates, cyclic voltammetry (CV) was performed in non-faradic regions at 30, 50, 70, 90, 110, and 130 mV s^−1^. Electrochemical impedance spectroscopy (EIS) was used to evaluate catalyst charge transport activity at applied onset potential of OER and an amplitude of 5 mV. Frequencies were between 100 000 Hz and 0.1 Hz. The Nernst equation was used to convert the measured potential into a reversible hydrogen electrode (RHE).

## 3. Results and discussion

### 3.1. Structural, compositional, and morphological characterizations

**Fig. 1:**
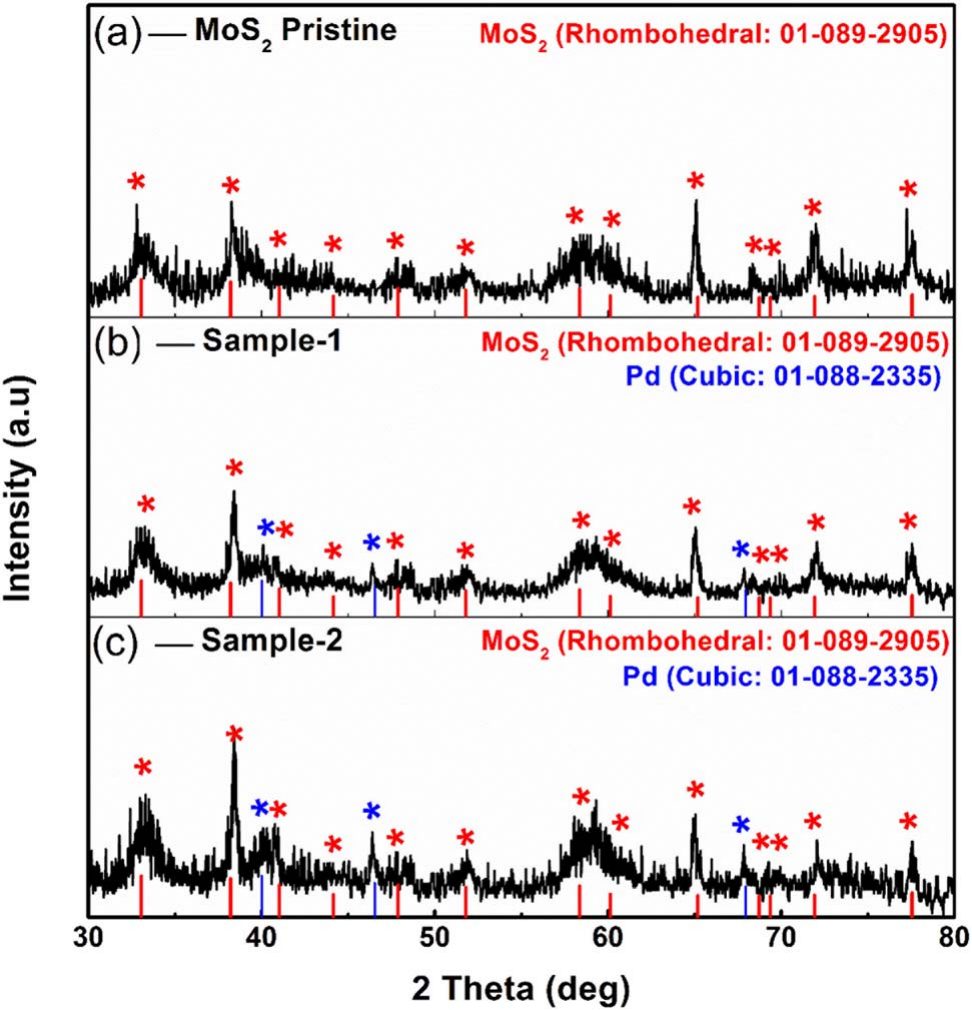
Powder XRD diffraction of (a) MoS_2_ pristine (b, c) sample-1 & sample-2.

Fig. 1 shows measured reflections for pristine MoS_2_ and Pd-doped MoS_2_, respectively, using powder XRD. As shown in Fig. 1, pristine MoS_2_ displayed significant crystalline properties and exhibited a variety of diffraction patterns. In the pristine MoS_2_, XRD diffractions at two theta angles were 33.065°, 38.209°, 41.028°, 44.142°, 47.878°, 51.799°, 58.357°, 60.133°, 65.184°, 68.735°, 69.377°, 71.918°, and 77.549° corresponding to Miller indices (101), (104), (015), (009), (017), (018), (1111), (021), (202), (024), and (0015), respectively. XRD analysis confirms that MoS_2_ is rhombohedral, as supported by JCPDS card no. 01-089-2905. The Pd based samples showed similar diffraction patterns as well as some reflections of nanoparticles of Pd. It was found that the reflections of the Pd nanoparticles were in good agreement with the standard JCPDS card no: 01-088-2335.^41^ Based on the measured reflections of Pd nanoparticles, they were indexed to 2 theta angles of 40.01°, 46.535°, and 67.925°. In the XRD study, it is apparent that MoS_2_ is a highly crystalline material and that Pd based composite has been confirmed, but there are no additional peaks corresponding to any impurities.

**Fig. 2:**
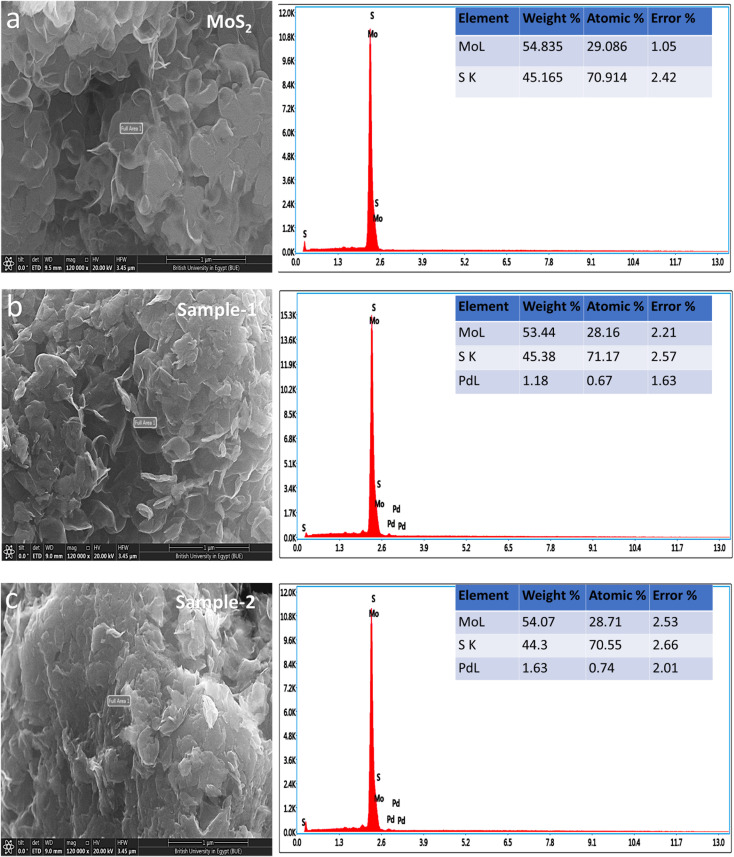
SEM and EDS spectra of (a) MoS_2_ pristine (b, c) sample-1 & sample-2.

Detailed morphology and chemical compositions of pristine and Pd based MoS_2_ composites are illustrated in Fig. 2 by SEM and EDS. According to Fig. 2, the left side shows the morphological features, while the right side shows the chemical compositions. In all cases, MoS_2_ exhibited sheet nanostructures with a thickness of 100–150 nm,^42^ and UV-light assisted Pd combination did not change this morphology. MoS_2_ samples were all uniformly morphologically characterized. Fig. 2 shows that the pristine sample of MoS_2_ contains Mo and S. However, EDS spectra of sample 1 and sample 2 clearly show the presence of Pd signals, confirming the presence of Pd. In comparison with sample 1, sample 2 has a relatively higher content of Pd according to the EDS study. Pd decorated MoS_2_ nanostructures was our goal with the intention of proposing an efficient electrocatalyst with a reasonable cost for large-scale applications using MoS_2_. In addition, EDS mapping as shown in Figure S1† confirms the even distribution of Mo, S and Pd.

**Fig. 3:**
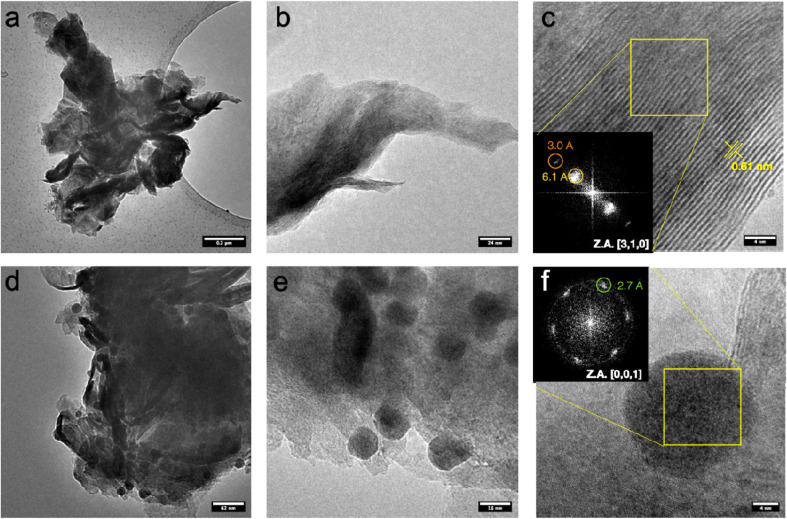
TEM micrographs a different magnification of pristine MoS_2_ (a–c) and PdNPs@MoS_2_ (d–f). (c, e) HRTEM micrographs with inset illustrating the spatial frequencies of the crystallite displayed in the FFT.

Fig. 3a–c shows TEM micrographs of pristine MoS_2_ at different magnifications. The samples are characterized by aggregated micro-flakes that have an average lateral size of over one micrometer.^42^ Apparently, the thickness of the nanosheets is quite dishomogeneous, ranging between few layers to 10–20 layers, as evidenced by the lattice fringes corresponding to MoS_2_ (0,0,2) planes on folded edges (Fig. 3c). A series of HAADF-STEM micrographs are shown in Fig. S2,† displaying a specific microstructure resulting from the folding of the nanosheets.

Fig. 3d–f shows TEM micrographs of MoS_2_ sample with Pd at different magnifications. Samples typically consist of micro-flakes with an average dimension of over 1 micrometer, like pristine MoS_2_ samples.^43^ However, nanoparticles with an average size ranging between 15 and 20 nm are observed to be deposited on MoS_2._ No specific crystalline feature related to these particles was observed, as evident in high magnification micrograph (Fig. 3f) where the only lattice fringes are compatible with MoS_2_, rather than metallic Pd. This is suggesting a low degree of crystallinity for the metallic nanoparticles, maybe resulting from partial oxidation. However, we cannot exclude that the lattice fringes related to the metal are not imaged as a result of the non-optimal imaging conditions, given the high thickness of the sample and the low operating acceleration voltage, limiting the resolution with such a low d-spacing, as the one expected for crystalline Pd (2.24 Å for (1,1,1) reflection).^44^ HAADF-STEM micrograph at different magnifications can be found in Fig. S3,† highlighting the higher contrast of the nanoparticles compared to the supporting MoS_2_ nanosheets. As contrast in this imaging mode is highly dependent on atomic weight *Z*, this is suggesting that the nanoparticles are Pd-based. STEM-EDS analysis (Fig. S4†) further corroborates these findings, display an increasing content of Pd on the nanoparticles. Strikingly, the Pd content registered on nanoparticles-free areas is not negligible, suggesting that Pd may be either adsorbed, or even embedded in the MoS_2_ lattice.

**Fig. 4:**
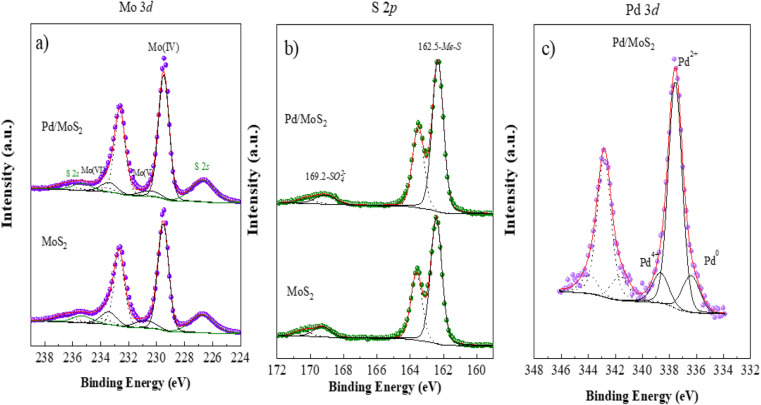
XPS spectra of pristine MoS_2_ and Pd doped sample-2 (a) Mo 3d, (b) S 2p, (c) Pd 3d.

The electronic environment and valence states of pristine MoS_2_ and Pd based MoS_2_ composite (sample-2) were evaluated using X-ray photoelectron spectroscopy (XPS). As shown in Fig. 4a, Mo 3d signals at the core level for pristine MoS_2_ and sample-2 evidence the presence of three different oxidation states for these samples with the Mo 3d_5/2_ contributions located at 229.5 eV, 230.8 eV and 233.4 eV, and assigned to Mo(iv), Mo(v) and Mo(vi), respectively.^45^ Moreover, it is observed two S 2s peak contributions.^46^ Comparing the sample with and without Pd, it is evidenced that under the presence of Pd, a greater proportion of Mo(iv) is present, 82% *vs.* 74%.

Fig. 4b shows the S 2p spectrum for both pristine MoS_2_ and Pd based sample-2, where the presence of both sulfide and sulfate species are clearly noticeable with the S 2p_3/2_ contribution centered at 162.5 eV and 169.2 eV, respectively. Again, the proportion of sulfide species is higher under the presence of Pd as observed for Mo signal, where the proportion of Mo(iv) is higher.^47^ Finally, Pd spectrum for Pd based MoS_2_ (sample-2) is shown in Fig. 4c. Three doublets, Pd 3d_5/2_ and 3d_3/2_ spin orbit contributions, are noticeable, with the Pd 3d_5/2_ component located at 336.4 eV, 337.6 eV and 338.7 and due to the presence of Pd^0^, Pd(ii) and Pd(iv), respectively.^48^ We have observed from the presence of Pd metallic nanoparticles into MoS_2_ using XRD analysis and the XPS study has shown that the Pd was located on the surface of MoS_2_ with various oxidation states such as Pd^0^, Pd^2+^ and Pd^4+^, and more likely the dominance is shown by the possible presence of PdO compared to PdO_2_ and Pd^0^ nanoparticles. The XRD has shown information about the material composition in bulk phase, whereas the XPS, being the surface science sensitive technique has given information about the chemical composition for less than 10 nm surface. Even though the presence of Pd in zero oxidation is less on the surface but it has indicated some support to the XRD analysis, hence we proposed the Pd^o^ nanoparticles decorated MoS_2_ composite throughout the text of manuscript.

### 3.2. OER half-cell water splitting performance of pristine MoS_2_ and various Pd based composites

**Fig. 5:**
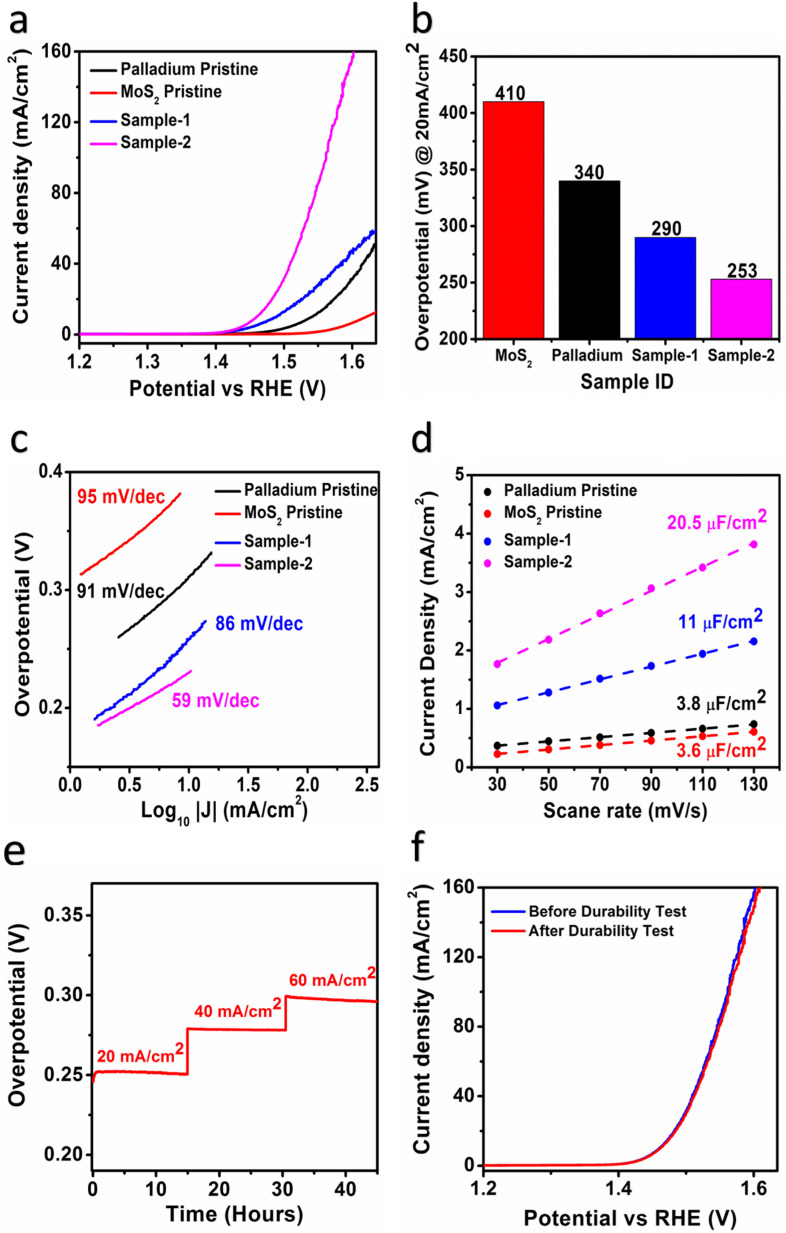
(a) LSV Polarization curves of different catalysts (b) bar graph presentation of different materials (c) corresponding Tafel plots for the different materials obtained from their LSV curves (d) *C*_dl_ values assigned to ECSA values (e & f) chrono-potentiometric durability at different current densities (20, 40 & 60 mA cm^−2^) of sample-2 and LSV curves before and after durability illustrating stability sample-2.

An electrochemical approach was used to investigate the role of OER in Pd based MoS_2_ samples under alkaline 1.0 M KOH conditions. In this preliminary OER characterization, linear sweep voltammetry (LSV) was used at a scan rate of 5 mV s^−1^ using a three-electrode cell setup. In this experiment, a modified glassy carbon electrode (GCE) was used with various samples of MoS_2_ as the working electrode, a reference electrode of silver–silver chloride (Ag/AgCl) saturated with 3.0 M KCl as the reference electrode, and a counter electrode of Platinum wire. We report all LSV polarization curves with *iR* corrections. According to Fig. 5a, LSV polarization curves were measured for different MoS_2_ samples and palladium particles in 1.0 M KOH, and measured curves showed that there were significant differences in OER activity among the samples. As pristine MoS_2_ has limited OER activity, various synthetic strategies for its structural and electronic disorder are necessary to enhance its performance. Palladium nanoparticles, however, were found to be even less effective in enhancing OER performance.^49^ These observations suggest that MoS_2_ being a low-cost, earth abundant material, and ecofriendly in nature could be commercialized if the efficient electrochemical water splitting performance would be obtained by improving the OER activity using dynamic synthesis strategies. In order to improve OER performance, we used a simple UV light assisted approach on hydrothermally grown MoS_2_ to couple with the minimum amount of palladium nanoparticles. Interestingly, using UV light as a light irradiation time provides us with control over palladium nanoparticle addition to MoS_2_. As illustrated in Fig. 5a, both PdNPs@MoS_2_ samples demonstrate significant enhancement of OER activity over bare palladium nanoparticles and pristine MoS_2_ samples. The Pd based MoS_2_ sample-2 has shown the lowest possible OER onset potential compared to other samples, suggesting the rare addition of palladium nanoparticles can lead to highly improved OER activity due to the increased electrical conductivity, rich catalytic sites, defects in the electronic structure, and synergetic effects between MoS_2_ and palladium materials through better interfacial charge transport.^50,51^ Furthermore, an overpotential at 20 mA cm^−2^ was estimated for the different samples as enclosed in Fig. 5b and it is obvious that pristine MoS_2_ sample was found with the highest OER overpotential of 410 mV, suggesting that it is limited by the sluggish reaction kinetics due to high energy barrier. Among the MoS_2_ samples, sample-2 showed the lowest overpotential of 253 mV and comparative analysis with other catalyst is mentioned in Table 1. In addition, it was observed during experimental results (Fig. S5†) that the addition of higher content of Pd (sample-3) led to deterioration of its reaction kinetics and decrease its OER performance. This might be that sample-3 had unfavorable surface and variable particle size of Pd nanoparticles which could not further support OER activity significantly. This indicates that the palladium incorporation has significantly lowered the energy demand of MoS_2_ towards OER process. Fig. 5c shows the corresponding Tafel values for each material based on Tafel analysis of the linear region of LSV curves. As shown in Fig. 5c, samples-2 and sample-1, palladium nanoparticles, and pristine MoS_2_ all showed calculated Tafel values in the order 59 mV dec^−1^, 86 mV dec^−1^, 91 mV dec^−1^, and 95 mV dec^−1^. It is evident from Tafel results that sample-2 of MoS_2_ has provided several channels for rapid OER kinetics, which validates the practical aspects of electrochemical water splitting.^39,52^

**Table 1:** Comparative study of PdNPs@MoS_2_ nanocomposite as OER catalyst with freshly reported electrocatalysts in 1 M KOH electrolyte

Electrocatalyst	Current density (mA cm^−2^)	Overpotential (mV)	Tafel slope (mV dec^−1^)	Ref.
Pd@MoS_2_	20	253	59	This work
NiCo_2_S_4_/C	20	285	61	58
Co_3_O_4_–MgO (CM)	10	274	64	54
Co_3_O_4_/CeO_2_	10	270	60	59
Co_2_P/CoNPC	10	311	78	60
TiO_2_–Co_3_O_4_	10	270	60	61
CoPO	10	350	60.7	62
MnO_2_@Co_3_O_4_	20	310	72	63
AMO@CeO_2_/NF	10	261	73.2	64
10-Ir-NCO	10	303	78	65

The OER kinetics for active sites involves four electron transfer steps, *i.e.*, OH^−^ exhibits coordinated oxidative adsorption on surface O vacancy sites (Eq. (1)). The adsorbed *OH is then oxidatively deprotonated to create *O (eqn (2)). In the subsequent O–O bond formation phase, *O interacts with another OH to generate a *OOH intermediate (eqn (3)). In the final phase, *OOH is deprotonated to produce O_2_ with the regeneration of the active site (eqn (4)).

1 OH^−^ → *OH + e^−^

2 *OH → *O + H^+^ + e^−^

3 *O + OH^−^ → *OOH + e^−^

4 *OOH → O_2_ + H^+^ + e^−^

whereas * represent the adsorbed states or adsorption active sites.^53^

**Fig. 6:**
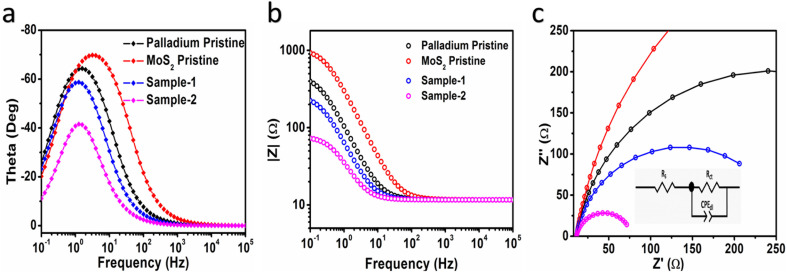
EIS experiment data of MoS_2_ pristine, different PdNPs@MoS_2_ like sample-1 and sample-2 at OER onset potential, amplitude of 10 mV for the frequency range of 100 kHz to 0.1 Hz in 1.0 M KOH (a, b) Bode plots and (c) Nyquist plot.

Based on cyclic voltammetry, we calculated electrochemical active surface area (ECSA) of sample-2 of MoS_2_ under a non-faradaic region at various scan rates as shown in Fig. S6.† Following that, a linear plot was constructed of the difference between the current densities of the anodic and cathodic sides *versus* the scan rate, as shown in Fig. 5d. As described in previous studies,^38,40,54^ the slope obtained after linear fitting corresponds to the ECSA value. In order, sample-2, sample-1, palladium nanoparticles, and pristine MoS_2_ have ECSA values of 20.5, 11, 3.8, and 3.6 μF cm^−2^ respectively. According to the ECSA calculations, sample-2 of MoS_2_ was highly likely to expose the large number of active sites during water splitting, resulting in enhanced OER activity compared to other materials used. As shown in Fig. 5e and f, we also examined the durability and stability aspects of sample-2 of MoS_2_ using chronopotentiometry and LSV polarization curves. The durability test was done at three different constant current densities like 20, 40, and 60 mA cm^−2^ as shown in Fig. 5e. Sample-2 exhibited excellent durability, and can be used for long-term water splitting measurements. Fig. 5f shows the LSV curves before and after the durability test, which confirms the sample-2 has high potential to maintain OER onset potential and overpotential without any abrupt changes.^51,55,56^ Based on the durability and stability results, MoS_2_ sample-2 can be used as an alternative to other existing precious and nonprecious electrocatalysts for OER. To obtain a comprehensive picture of the enhanced OER activity of sample-2 of MoS_2_, electrochemical impedance spectroscopy (EIS) has been used to examine the interfacial charge transfer behavior of the various prepared samples in the present study, as shown in Fig. 6. This was achieved by scanning different materials with a sweeping frequency of 100 000 Hz to 1 Hz, amplitude of 5 mV and OER onset potential of 0.45 V, including pristine MoS_2_, Pd doped samples-1 and 2, and palladium nanoparticles. As shown in Fig. 6a–c, Bode plots and Nyquist plots have been used to represent the experimental EIS data. Charge transports as determined by Nyquist plots have been strongly supported by Bode plots. Simulation of experimental EIS data with z-view software led to a well fitted equivalent circuit, as shown inset in Fig. 6c. Essentially, the equivalent circuit consisted of the constant phase element (CPE), charge transfer resistance (*R*_ct_), and solution resistance (*R*_s_).^31,57^ In Table 2, we present the estimated values for charge transfer resistance. In comparison with other materials reported in this work, sample-2 had the lowest charge transfer resistance, which confirms that palladium incorporation into MoS_2_ has enhanced sample-2's electrical conductivity, which has further contributed to the efficient operation of OERs. Moreover, we have compared the obtained results of sample-2 with the recently published works on the OER applications as given in Table 1. It can be seen that the newly designed electrocatalyst based on PdNPs@MoS_2_ (sample-2) has several advantages such as low overpotential, simple, and ecofriendly, hence it can be used as an alternative electrode material for the energy conversion and storage applications.

**Table 2:** Summary of unique features of presented OER catalysts

Catalyst	Calculated from LSV	Calculated from EIS		Calculated from CV	
	Tafel slope	Charge transfer resistance	Double layer capacitance	Double layer capacitance	Electrochemically active surface area
	*B*, mV dec^−1^	*R*_ct_, Ω	CPE_dl_, mF	*C*_dl_, μF cm^−2^	ECSA, cm^2^
Palladium pristine	91	465.6	1.75	3.8	95
MoS_2_ pristine	95	1038	0.56	3.6	90
Sample-1	86	251.7	2.90	11	275
Sample-2	59	65.55	5.18	20.5	512.5

## 4. Conclusions

In summary, PdNPs were incorporated onto hydrothermally grown MoS_2_ nanostructures using UV light to yield PdNPs@MoS_2_. The proposed approach for designing an efficient MoS_2_ based electrocatalyst employing structural changes is facile, scalable, and eco-friendly for hydrogen (H_2_) production. A variety of analytical techniques have been used to assess the structure, morphology, crystal quality, and surface chemical composition of the samples. UV light assisted incorporation of PdNPs did not change the morphology of MoS_2_, but altered its chemical composition as well as the electrochemical behavior to favor OER. In particular, sample-2 of PdNPs@MoS_2_, the OER performance has taken place at the lowest overpotential (253 mV at 20 mA cm^−2^) with significant stability and durability. Low charge transfer resistance for the prepared samples strongly supported the OER efficiency. Furthermore, the addition of Pd had not only optimized the concentration but also created adverse effect on the OER activity of the composite system. In light of the findings, the presented electrode material may have greater potential for energy conversion and storage applications.

## Conflicts of interest

Authors declare no conflict of interest in this research work.

## Data availability

The authors declare that the data supporting the findings of this study are available upon request.

## Author contributions

Umair Aftab, did material synthesis and partial electrochemical studies. Muhammad Yameen Solangi, did partial electrochemical investigations. Aneela Tahira, did XRD analysis and wrote the draft. Abdul Hanan, did ECSA study. Muhammad Ishaq Abro, did partial supervision. Amal Karsy, did SEM measurement. Elmuez Dawi, did validation of electrochemical results and edited the draft. Muhammad Ali Bhatti, did SEM and EDS analysis. Riyadh H. Alshammari, did EDS mapping and analyzed the results. Ayman Nafady, did EIS analysis and edited the draft. Alessandro Gradone and Raffaello Mazzaro, did TEM/HRTEM measurement, Vittorio Morandi, supervised the TEM/HRTEM measurement. Antonia Infantes-Molin, did XPS analysis. Zafar Hussain Ibupoto, did main supervision and wrote the first draft of manuscript.

## Supplementary Material

RA-013-D3RA04738E-s001
