# Supplementary material for: An advanced PdNPs@MoS2 nanocomposite for efficient oxygen evolution reaction in alkaline media
Source: RSC Adv. 2023 Nov 3;13(46):32413–23. doi: 10.1039/d3ra04738e (PMC10623383; doi:10.1039/d3ra04738e)
Supplement: RA-013-D3RA04738E-s001 [file RA-013-D3RA04738E-s001.pdf]

Electronic Supplementary Material (ESI) for RSC Advances.
This journal is © The Royal Society of Chemistry 2023

Supporting Information

An advanced PdNPs@MoS_2 nanocomposite for efficient oxygen evolution reaction in alkaline media

Umair Aftab[a], Muhammad Yameen Solangi[a], Aneela Tahira[b], Abdul Hanan[d], Muhammad Ishaq Abro[a], Amal Karsy[h], Elmuez Dawi[i], Muhammad Ali Bhatti[j], Ayman Nafady[g], Alessandro Gradone[f], Raffaello Mazzaro[f,l],, Vittorio Morandi[f], Antonia Infantes-Molin[e], Zafar Hussain Ibupoto[c]

[a]Department of Metallurgy and Materials Engineering, Mehran University of Engineering and Technology, 76080, Jamshoro, Pakistan.

[b]Institute of Chemistry, Shah Abdul Latif University Khairpur Mirs, Sindh, Pakistan.

[c]Dr. M.A Kazi Institute of Chemistry University of Sindh Jamshoro, 76080, Sindh Pakistan.

[d]Key Laboratory of Superlight Material and Surface Technology, Ministry of Education, College of Materials Science and Chemical Engineering, Harbin Engineering University, 150001, Harbin, PR China.

[e]Department of Inorganic Chemistry, Crystallography and Mineralogy. (Unidad Asociada al ICP-CSIC), Faculty of Sciences, University of Malaga, Campus de Teatinos, 29071, Malaga, Spain.

[f]Department of Science and Technology, CNR IMM, Bologna, Italy.

[g]Department of Chemistry, College of Science, King Saud University, Riyadh, 11451, Saudi Arabia.

[h]Nanotechnology Research Centre (NTRC), The British University in Egypt (BUE), Cairo, Egypt.

[i]Nonlinear Dynamics Research Center (NDRC), Ajman University, Ajman, P.O. Box 346, United Arab Emirates.

[j]Institute of Environmental Sciences, University of Sindh Jamshoro, Jamshoro, 76080, Sindh, Pakistan

Corresponding authors**: *Zafar Hussain Ibupoto,* ***Email: *zaffar.ibhupoto@usindh.edu.pk*

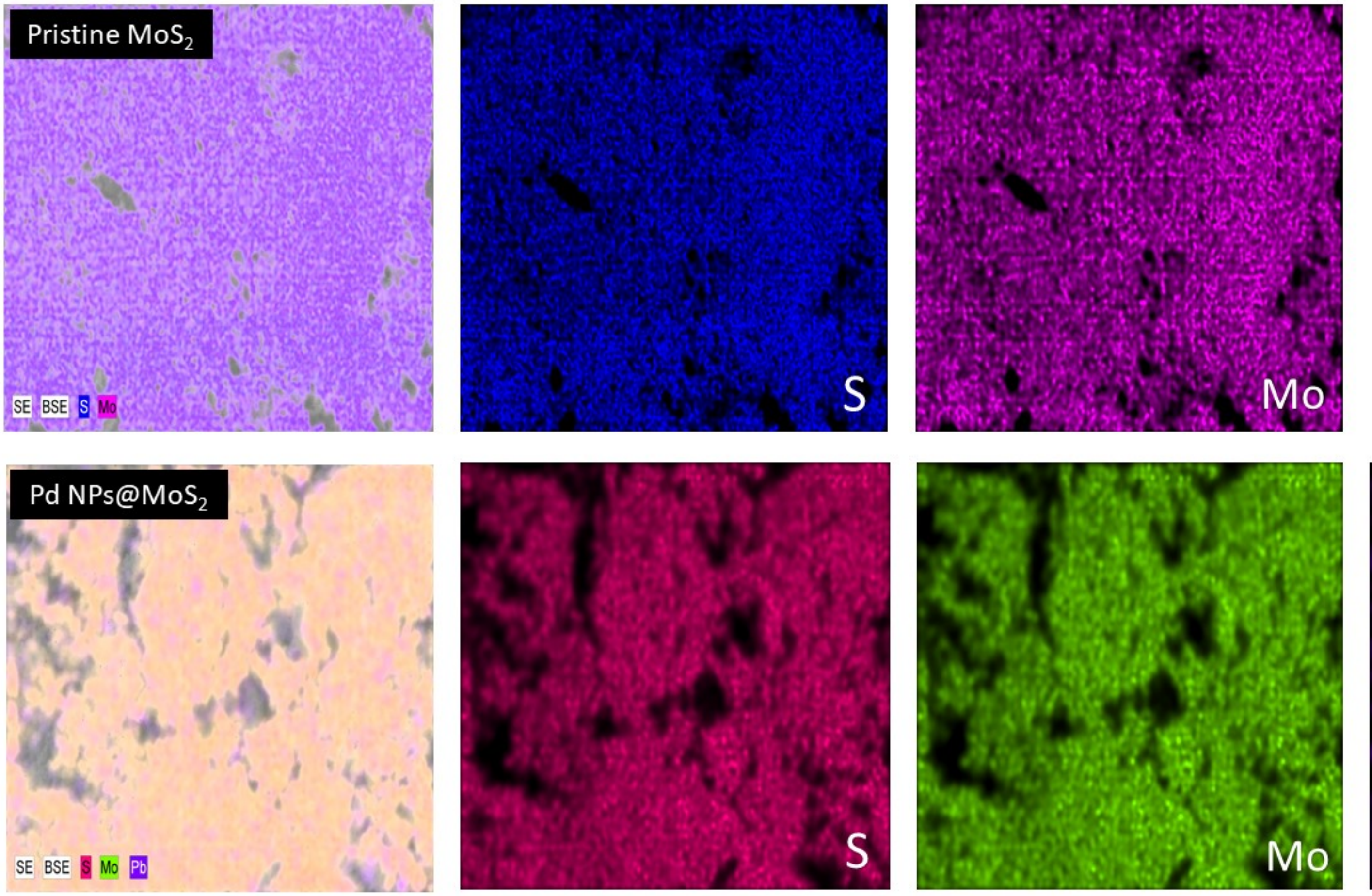

Fig S1: EDS Mapping of MoS_2 Pristine and Pd doped sample.

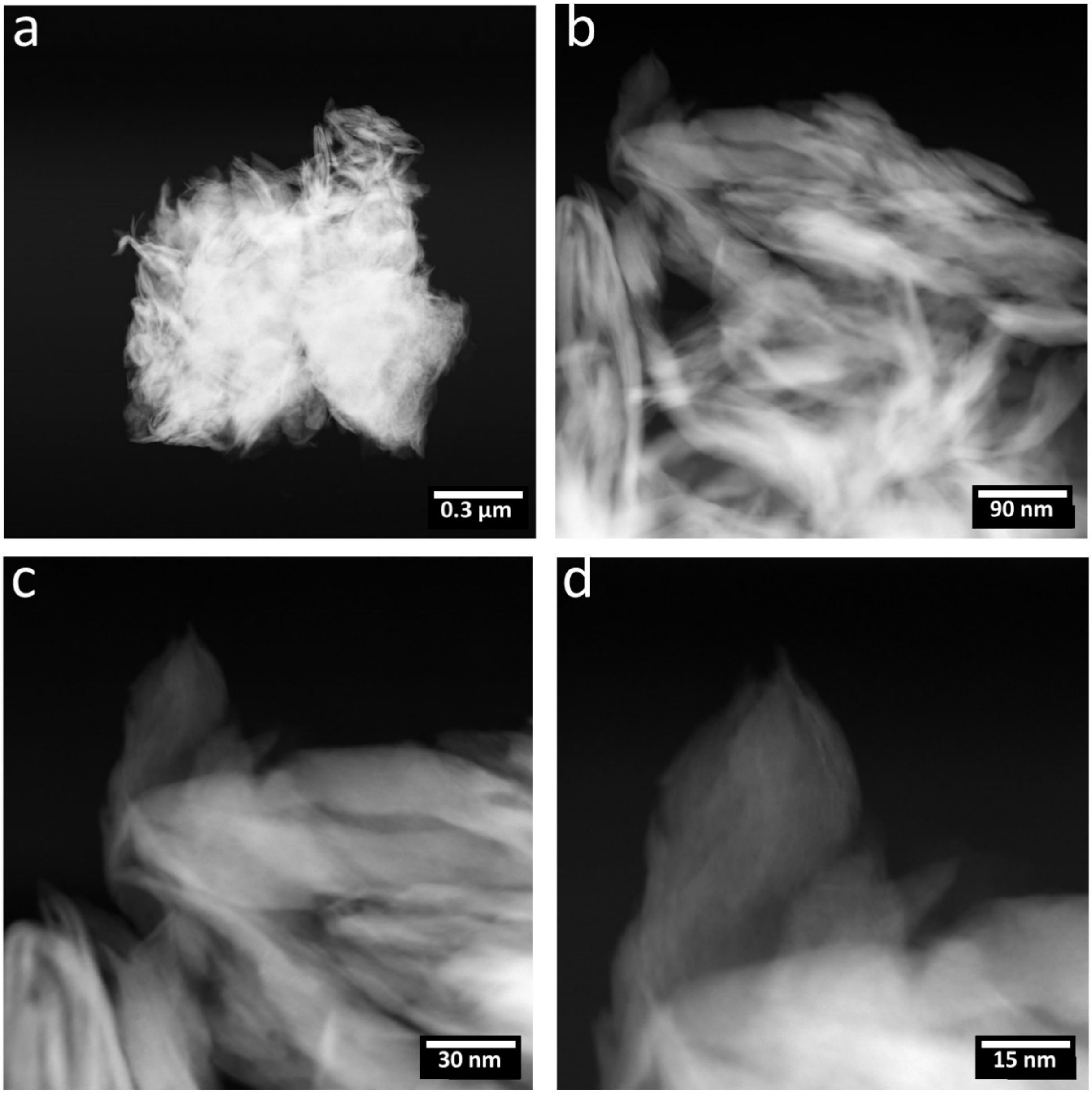

Fig S2: HAADF – STEM micrographs at different magnifications of MoS_2 pristine.

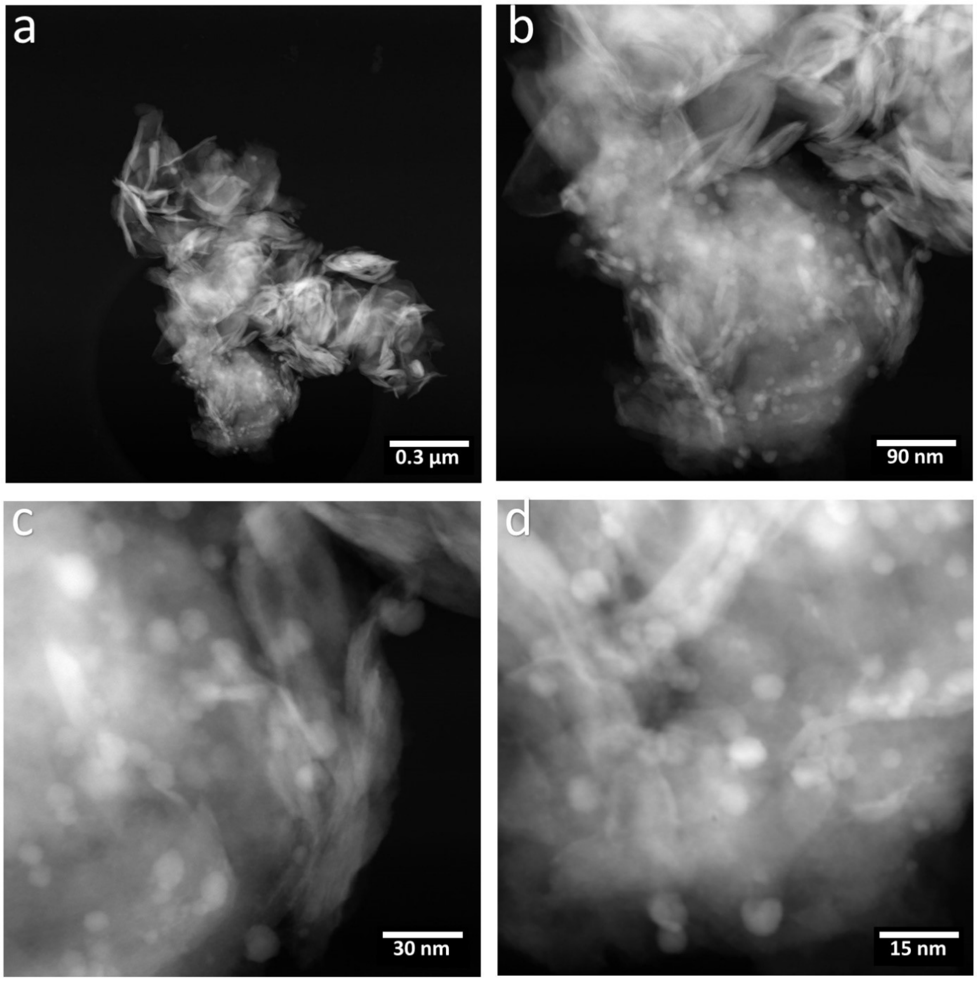

Fig S3: HAADF – STEM micrographs at different magnifications of Pd doped sample.

Comment [UdMO]: Please use always the same name. In this case $PdNPs@MoS_2$ was used.

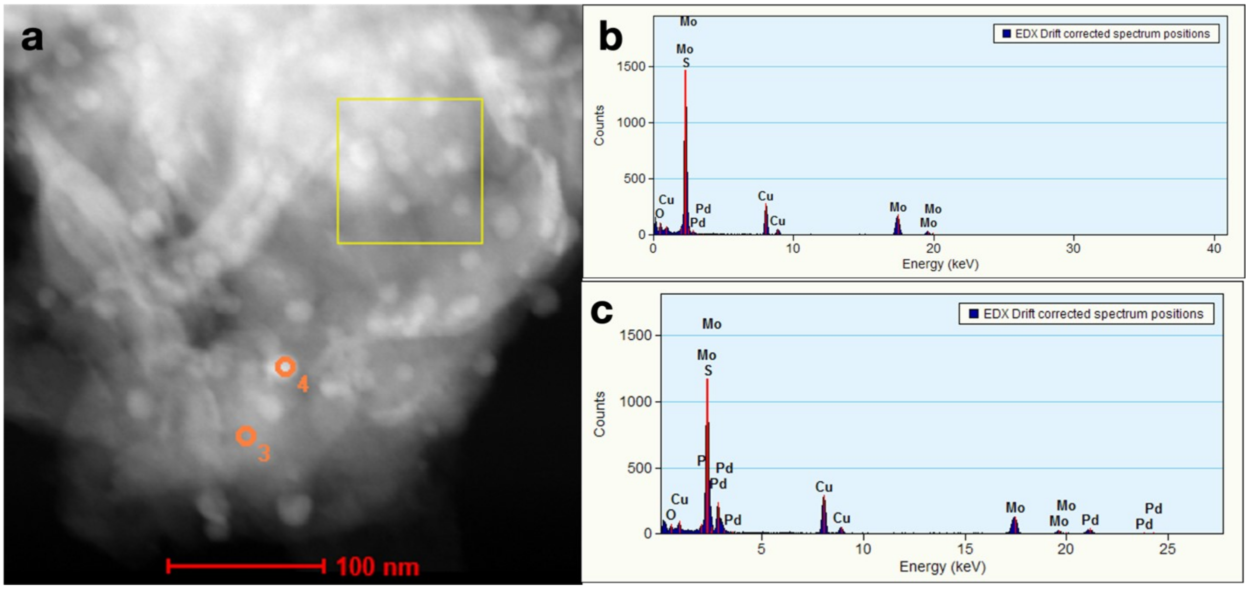

Fig S4. PdNPs@MoS_2 sample a) STEM-HAADF micrograph. b,c) EDS position analysis of the spot 3 (b) and spot 4 (c), highlighted in the STEM image.

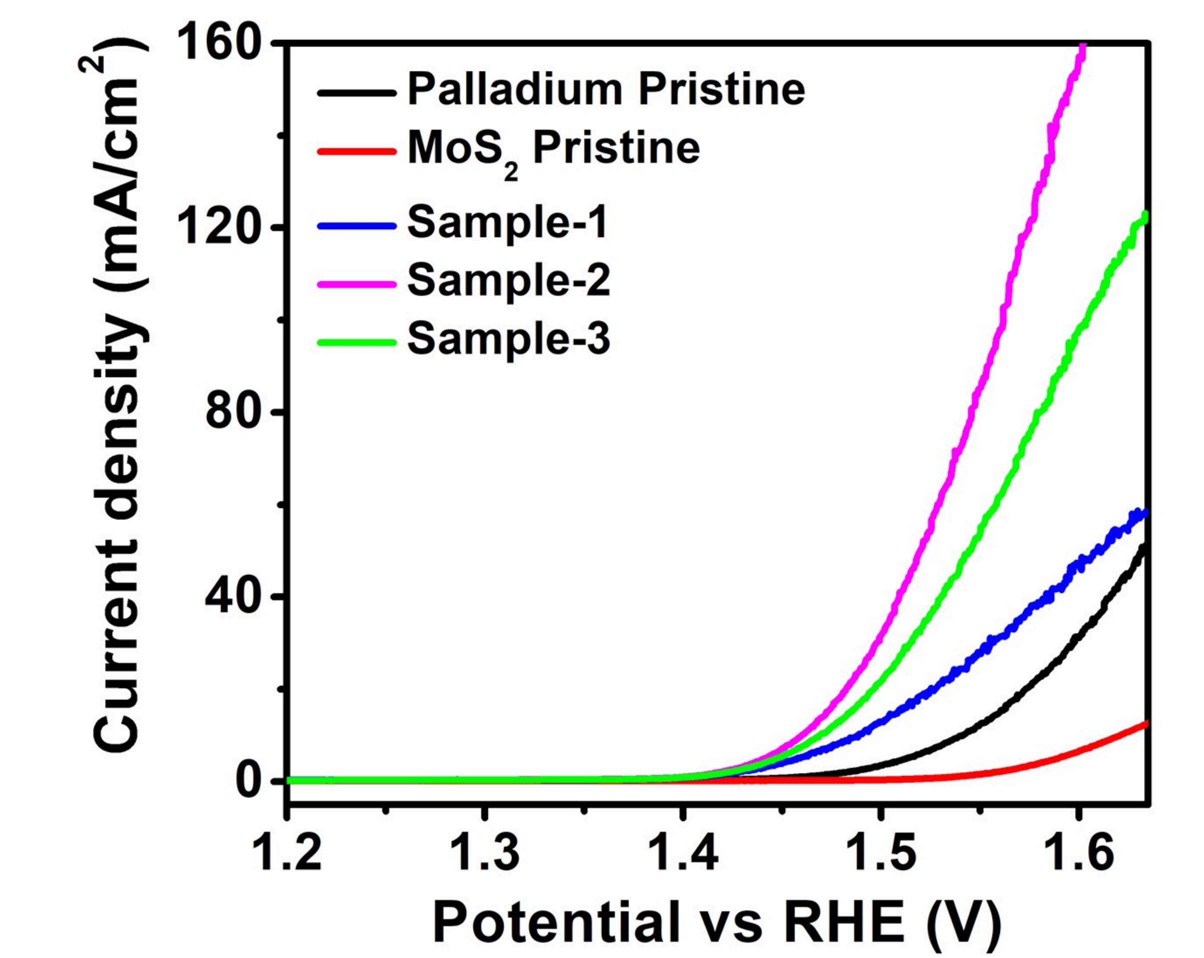

Fig S5. Polarization curve of different catalyst containing high concentration of Pd (Sample-3)

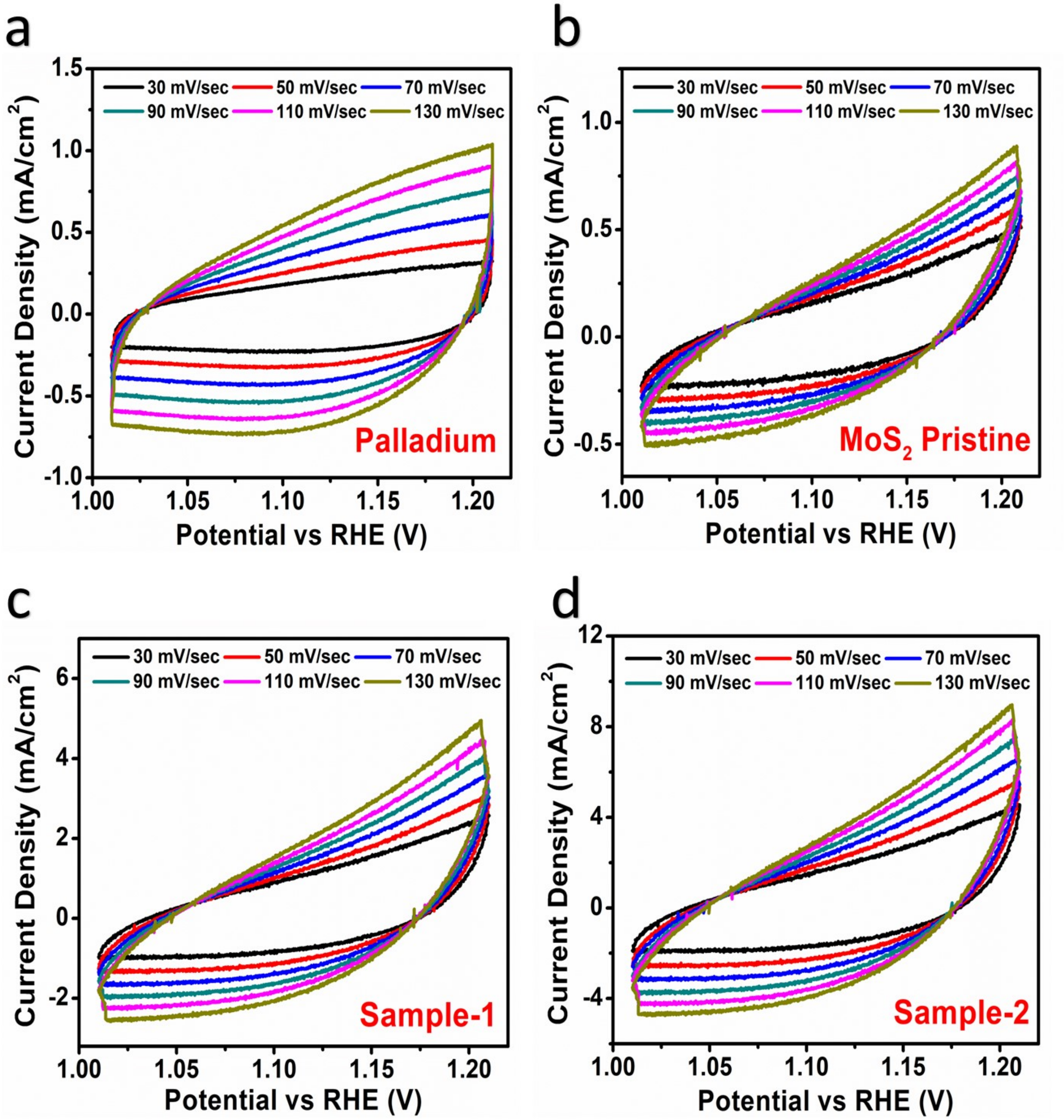

Fig S6: CV curves at various scan rates of (a) Palladium Pristine, (b) MoS_2 Pristine, (c, d) Sample-1 & Sample-2 for the determination of double layer capacitance.
